# Control of pre-replicative complex during the division cycle in *Chlamydomonas reinhardtii*

**DOI:** 10.1371/journal.pgen.1009471

**Published:** 2021-04-28

**Authors:** Amy E. Ikui, Noriko Ueki, Kresti Pecani, Frederick R. Cross

**Affiliations:** 1 Department of Biology, Brooklyn College, The City University of New York, New York City, New York, United States of America; 2 Laboratory of Cell Cycle Genetics, The Rockefeller University, New York City, New York, United States of America; Washington University School of Medicine, UNITED STATES

## Abstract

DNA replication is fundamental to all living organisms. In yeast and animals, it is triggered by an assembly of pre-replicative complex including ORC, CDC6 and MCMs. Cyclin Dependent Kinase (CDK) regulates both assembly and firing of the pre-replicative complex. We tested temperature-sensitive mutants blocking *Chlamydomonas* DNA replication. The mutants were partially or completely defective in DNA replication and did not produce mitotic spindles. After a long G1, wild type *Chlamydomonas* cells enter a division phase when it undergoes multiple rapid synchronous divisions (‘multiple fission’). Using tagged transgenic strains, we found that MCM4 and MCM6 were localized to the nucleus throughout the entire multiple fission division cycle, except for transient cytoplasmic localization during each mitosis. *Chlamydomonas* CDC6 was transiently localized in nucleus in early division cycles. CDC6 protein levels were very low, probably due to proteasomal degradation. CDC6 levels were severely reduced by inactivation of *CDKA1* (*CDK1* ortholog) but not the plant-specific *CDKB1*. Proteasome inhibition did not detectably increase CDC6 levels in the *cdka1* mutant, suggesting that CDKA1 might upregulate CDC6 at the transcriptional level. All of the DNA replication proteins tested were essentially undetectable until late G1. They accumulated specifically during multiple fission and then were degraded as cells completed their terminal divisions. We speculate that loading of origins with the MCM helicase may not occur until the end of the long G1, unlike in the budding yeast system. We also developed a simple assay for salt-resistant chromatin binding of MCM4, and found that tight MCM4 loading was dependent on ORC1, CDC6 and MCM6, but not on RNR1 or CDKB1. These results provide a microbial framework for approaching replication control in the plant kingdom.

## Author summary

The unicellular green alga *Chlamydomonas reinhardtii* undergoes cell division by ‘multiple fission’: repeated synchronous rounds of DNA replication and mitosis producing 8–16 daughter cells. In this study, we show that MCM proteins are localized to the nucleus throughout the multiple fission cycle, expect for transient loss during mitosis. CDC6 was also transiently localized to the nucleus. CDC6 levels were regulated by proteosomal protein degradation. We found evidence that the step-wise assembly of pre-replicative complex was conserved in *Chlamydomonas*. The plant-specific CDKB kinase was not required for this assembly. We propose a model that pre-RC assembly takes place right before the first replication cycle; assembly may be repeated in each cycle after the replication proteins are diffused in mitosis. This mechanism may contribute to timely replication in the multiple fission cycle.

## Introduction

The cell cycle is an ordered set of cellular processes in which DNA is replicated and segregated into two identical daughter cells. In many eukaryotic organisms, the cell cycle is controlled by cyclin dependent kinases (CDK), bound to cyclin activators [1]. *Chlamydomonas reinhardtii* is a single cell green algae which divides by ‘multiple fission’: a long G1 phase with massive cell growth, followed by typically 3–4 rounds of S/M phase that consists of DNA replication, chromosome segregation and cytokinesis [2]. The resulting daughter cells remain within the mother cell wall, finally hatching to produce 8–16 small newborn G1 cells that restart the cycle. There are two major CDKs in *Chlamydomonas reinhardtii*, *CDKA1* (the ortholog of animal *CDK1*) and *CDKB1* (a *CDK* specific to the plant kingdom) [3,4]. While *CDK1* is the direct trigger of mitosis in yeast and animals, *CDKA1* functions in early in the cell cycle and *CDKB1* promotes mitosis. One important role of *CDKA1* early in the cell cycle is transcriptional activation of a large battery of genes required for cell division that are turned on shortly before the division cycles begin [5,6].

DNA replication has been studied extensively in the budding yeast *S*. *cerevisiae*. To initiate DNA replication in yeast, ORC (Origin recognition complex) binds to origins followed by Cdc6 (Cell division cycle), Cdt1p and Mcm2-7 helicase (Minichromosome maintenance), forming the pre-replicative complex (pre-RC) [7–10]. At this stage, MCM2-7 complex is bound on replication origins in an inactive form which triggers origin licensing. CDK activity is required to phosphorylate multiple proteins to assemble a functional replisome which contains active helicase such as CDC45-MCM2-7-GINS which leads to firing of MCM-loaded replication origins [11]. At this stage, MCM2-7 are tightly bound and loaded on chromatin which are resistant to high concentration of salt wash *in vitro* [12]. Once DNA replication is initiated, the pre-RC components such as Cdc6, Mcm2-7, and the ORC complex are phosphorylated by Cyclin/CDK; these phosphorylations prevent a second round of MCM loading, resulting in precise alternation of DNA replication and mitosis, when CDK is inactivated and origins can reload [13]. For example, Cdc6 is phosphorylated by Cyclin/CDK, targeting it for ubiquitin-mediated proteolysis through the SCF complex [14–17], Mcm2-7 is exported from the nucleus through CDK-dependent phosphorylation on NLS/NES module [18], and ORC function is inhibited by phosphorylation. These mechanisms collectively prevent origin relicensing within S phase.

The pre-RC proteins are mostly conserved comparing yeast to humans, but there are significant functional differences. While ScOrc1-6 binds to specific origin DNA sequences throughout the cell cycle, HsOrc1-6 transiently associates with chromatin only during G1 phase, and without a defined origin consensus sequence [19]. HsMcm2 and HsMcm4 are phosphorylated by Cyclin B/Cdc2 during the G2-M phase [20]. These phosphorylations inhibit HsMcm2-7 reloading onto chromatin [21], but do not cause nuclear export as in yeast. HsCdc6 is localized to the nucleus in G1 with a small fraction of HsCdc6 in the cytoplasm during S phase [22]. SCF-dependent HsCdc6 degradation inhibits DNA re-replication [23]. In contrast to yeast, where CDK-dependent phosphorylation promotes Cdc6 proteolysis, CDK-dependent HsCdc6 phosphorylation prevents Cdc6 proteolysis [24,25].

ORC, CDC6 and CDT1 have been identified in the land plant *Arabidopsis thaliana* [26]. AtORC1b is expressed only in proliferating cells. AtCDC6 is expressed during S-phase under control of E2F dependent transcription [27]. Ectopic expression of AtCDC6 or AtCDT1 induces endoreplication, therefore proper Cdc6 expression and regulation is required to maintain genome integrity [28,29]. AtCDC6 is degraded in a cell-free system in a proteasome-dependent manner [29]. AtMCM2-7 was expressed preferentially in tissues where DNA replication was occurring and plays a role in seed development [30,31]. While AtMCM7 (PROLIFERA) is transiently localized to the nucleus of root meristem cells during G1 [32], AtMCM5 and AtMCM7 localize to the nucleus in G1, S and G2 phases and only transiently translocate to the cytoplasm during mitosis in transgenic tobacco cells [30]. In maize, MCM6 is expressed at its highest level in the nucleus during G1 and then translocates to the cytoplasm during S phase [33]. Therefore, the function of MCM2-7 in plants may be regulated by localization, though not in a uniform fashion across species. Plant *ORC*, *CDC6*, *CDT1* and *MCMs* have been shown genetically to be essential; however, the absence of conditional mutant alleles has made it difficult to functionally address mechanisms of DNA replication control at the level of single cells in the plant kingdom [34].

DNA replication control has been little studied in *Chlamydomonas*. In this study, we analyzed DNA replication mutants identified in our previous UV-mutagenesis screening in *Chlamydomonas reinhardtii* [35,36], concentrating on pre-RC components and their relationship to CDKA1 and CDKB1 activity.

## Results

### Completion of the first cycle of DNA replication and nuclear division are inhibited in DNA replication mutants

**Fig 1:**
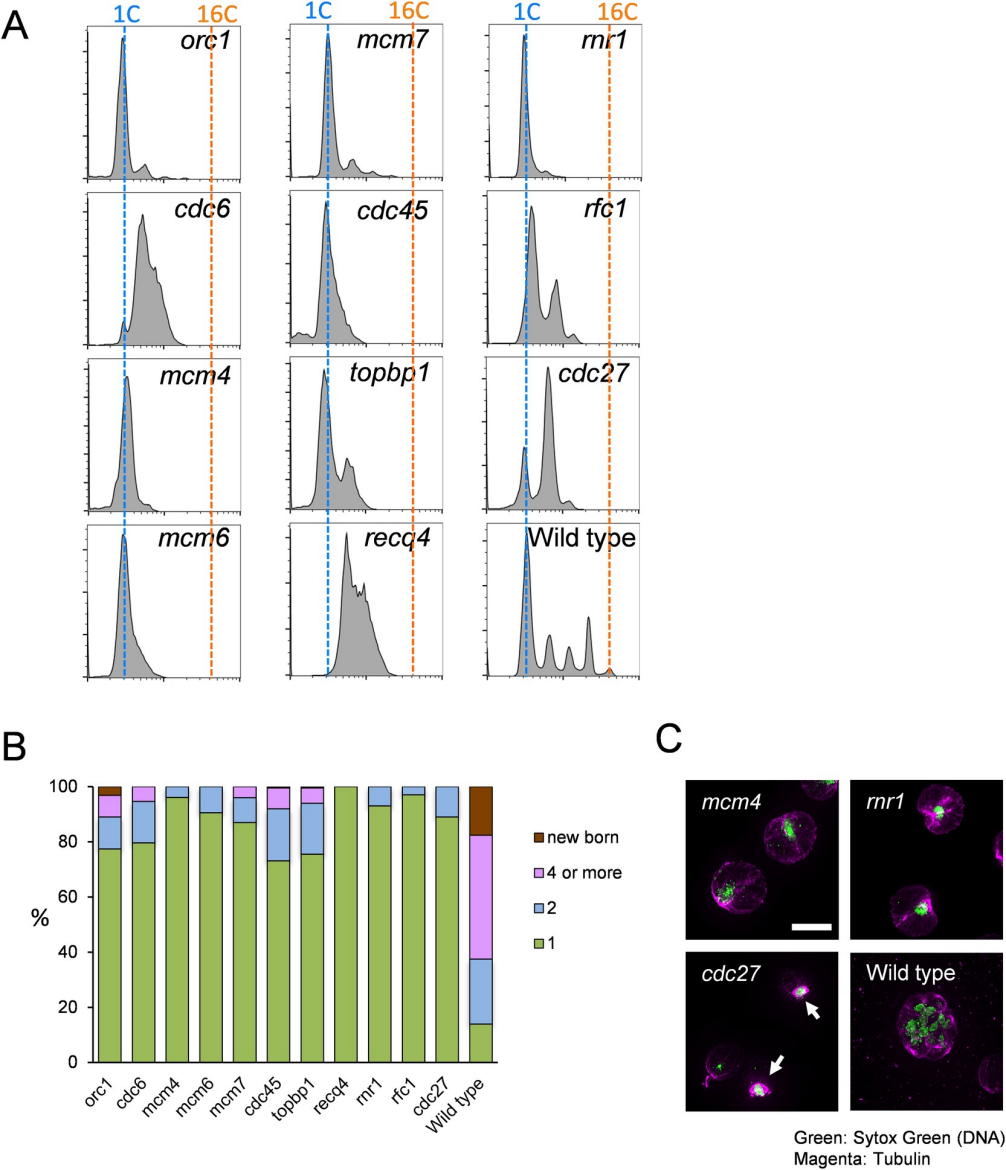
Analysis of DNA replication mutants (A) Wild type cells were arrested in G1 by nitrogen starvation and the cell cycle was released for 12 hrs at the non-permissive temperature 33°C. Cells were fixed and stained with Sytox for FACS. (B) The number of cells with newborn, 4 or more nuclei, 2 nuclei or 1 nucleus were counted using the samples from A. 100 cells were counted and the percentage is shown. (C) Indicated temperature sensitive mutants and Wild type cells were arrested in G1 and the cell cycle was released for 12 hrs at 33°C. Cells were fixed and stained with anti-tubulin antibody (Magenta) and Sytox Green (Green). Images were taken by DeltaVision microscope. White arrows show mitotic spindles. Bar = 10 μm.

In order to monitor cell cycle progression and to obtain a synchronous cell population in *Chlamydomonas reinhardtii*, we studied cells synchronized by nitrogen deprivation (causing arrest in G1) and refeeding. At intervals, cells were collected, fixed and stained with Sytox followed by flow cytometry analysis (FACS). By 12–14 hrs after release, wild type cells undergo multiple synchronous cycles of DNA replication and mitosis within the mother cell wall, yielding multinucleate cells producing signals of DNA content of 1, 2, 4, 8 and 16C (Fig 1A and 1B). Chloroplast DNA replication has been reported to be uncoupled from the nuclear replication cycle, and likely only accounts for a small part of the total FACS signal [37].

The DNA replication mutant cells were defective to varying degrees. Some showed no detectable increase in DNA content; others appeared to complete most of the first DNA replication cycle. However, all mutants were severely defective in nuclear division and entry into a second round of DNA replication (Fig 1A and 1B). Thus, DNA replication is inhibited in the temperature sensitive (ts) DNA replication mutants as expected; in addition, incomplete DNA replication in the first cycle may block both nuclear division and re-initiation for a second round. Lack of nuclear division correlated with absence of detectable mitotic spindle formation by anti-tubulin immunofluorescence (Fig 1C). In yeast and animals, incomplete replication similarly triggers efficient blocks to mitotic progression [38].

### Synthetic lethality between pre-RC mutants

**Fig 2:**
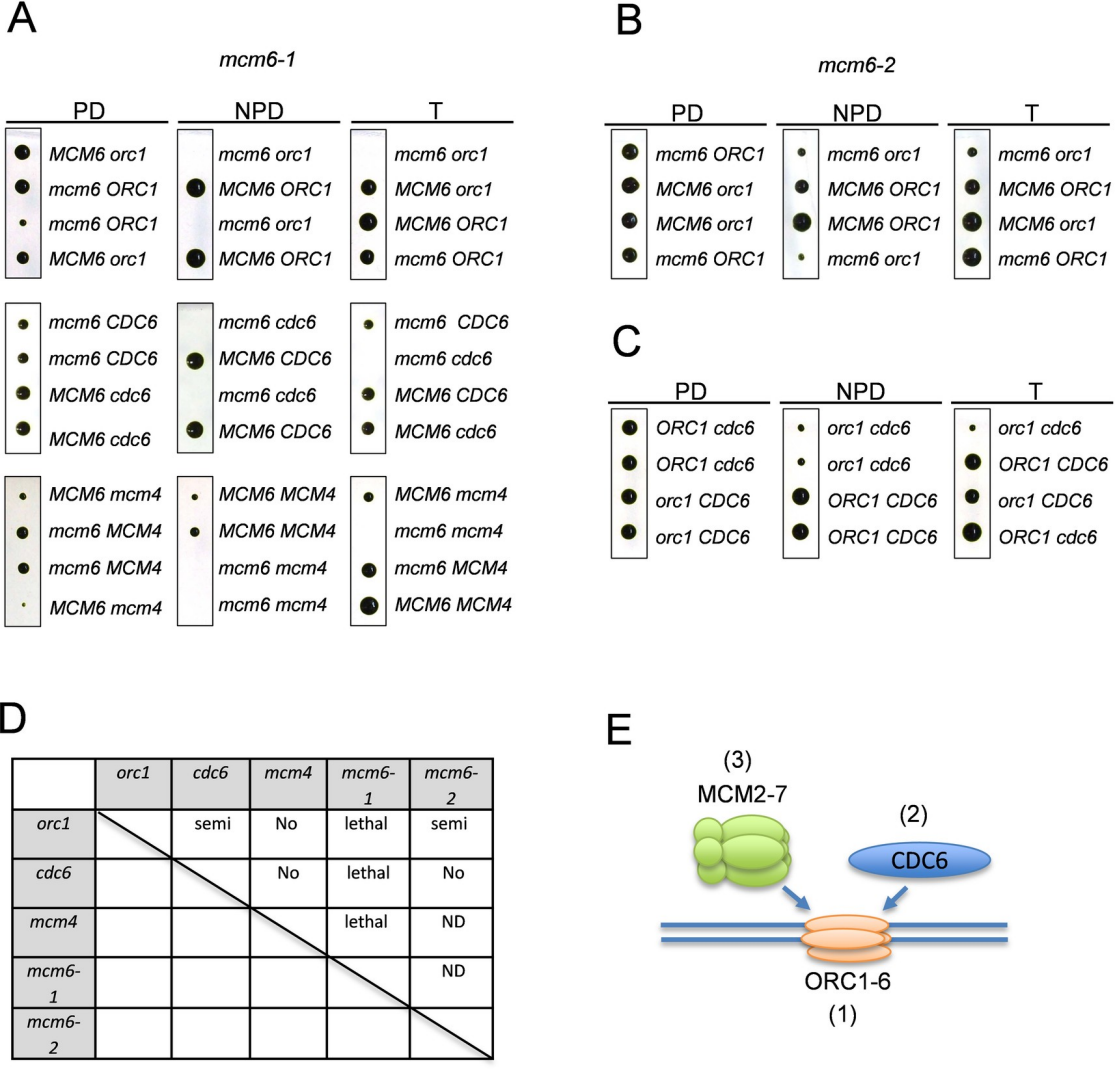
Synthetic lethality between DNA replication mutants (A) *mcm6-1* temperature sensitive mutant was crossed with *orc1-1*, *cdc6-1* or *mcm4-1* mutant. Tetrads are shown with an example of parental ditype (PD), non-parental ditype (NPD) or tetra type (T). (B) Tetrad analysis of *mcm6-2 orc1-1* and (C) *orc1-1 cdc6-1* are shown. (D) A summary table of synthetic lethality tested in this study. Lethal: complete lethal, Semi: mild lethality, No: No genetic interaction, ND: No data. (E) A diagram of pre-RC components. The number shows the order of the protein binding to DNA in other eukaryotes.

If *Chlamydomonas* pre-RC components function in complexes, as do their yeast and human orthologs, then strong genetic interactions might be expected between mutations in different genes in this set (Fig 2E). We inter-crossed pre-RC mutants *orc1-1*, *cdc6-1*, *mcm4-1*, *mcm6-1* and *mcm6-2* (previously named *mcm6-981A* and *mcm6-GHI* [36]) and analyzed viability of double mutant progeny at the permissive temperature. *mcm6-1* and *mcm6-2* contain distinct mutations (S1 Table). *mcm6-1* was synthetically lethal with *orc1-1*, *cdc6-1* and *mcm4-1* (Fig 2A). *mcm6-2* showed a mild deleterious interaction with *orc1-1* (Fig 2B). Combination of *orc1-1* and *cdc6-1* was similarly mildly deleterious (Fig 2C). The genetic interactions are summarized in Fig 2D. Frequent deleterious interactions in this mutant set support the idea that these proteins form functional complexes, as expected from prior work in yeast (see Introduction). This genetic result also confirms the interactome network of DNA replication proteins in *Arabidopsis* [39].

### Transgenes expressing fluorescent fusion proteins rescued temperature sensitivity of the replication mutants

We created universal tagging plasmids containing mCherry, Venus or GFP to be used in *Chlamydomonas* (S1 Fig). We amplified *CDC45* PCR product from genomic DNA and inserted it into the mCherry plasmid through Gibson assembly. The resulting *CDC45-mCherry* plasmid was linearized with XbaI and electroporated into the *cdc45* temperature sensitive mutant. Transformants were selected for antibiotic resistance at 21°C (permissive temperature) and then screened at the non-permissive temperature, 33°C (S2 Fig). A small proportion of transformants grew at 33°C; such low co-rescue is a general feature of *Chlamydomonas* transformation, probably resulting from frequent breakage or silencing of transforming DNA. Colonies that grew at 33°C were picked and their genomic DNA was tested by PCR for the presence of mCherry, the region covering the promoter and the ampicillin resistance gene. No temperature-resistant colonies were obtained with the empty vector (S2 Fig).

**Fig 3:**
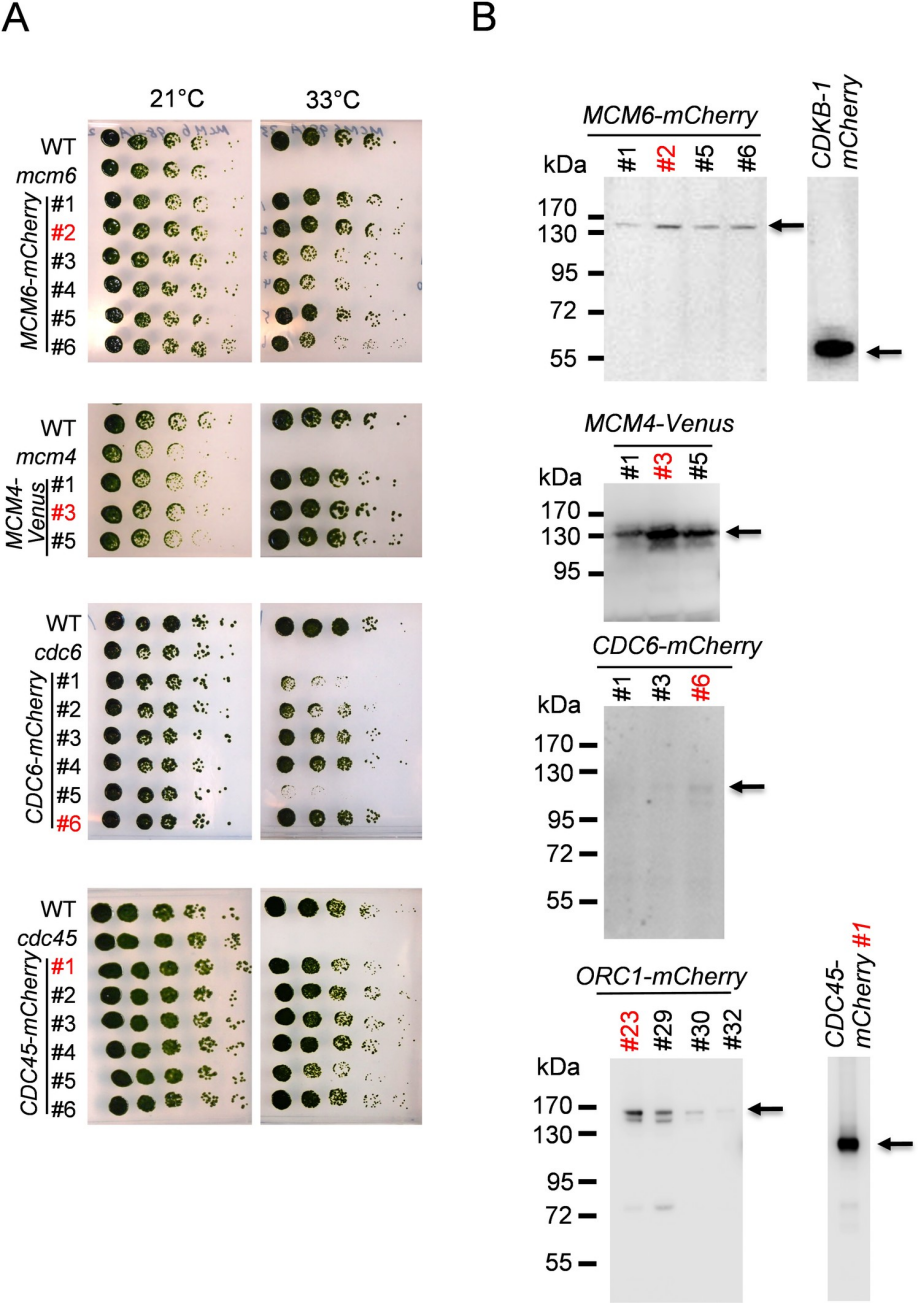
Transgene of *MCM6-mCherry*, *MCM4-Venus*, *CDC6-mCherry*, *CDC45-mCherry* rescued temperature sensitivity of the corresponding mutants. (A) Indicated strains were grown and serially diluted 5-fold on TAP plates. The plates were incubated at 21 or 33°C for 5–7 days. The strain number in red was used in the following experiments. (B) Protein expression of MCM6-mCherry, MCM4-Venus, CDC6-mCherry, ORC1-Venus and CDC45-mCherry were detected by western blotting using anti-mCherry or anti-GFP antibody. CDKB1-mCherry is shown as a positive control.

Similarly, we constructed tagging plasmids to rescue other ts-lethal mutants. Efficiency of rescue in the selected transformants was confirmed by serial dilution (Fig 3A). Selected transformants were further subjected to western blotting to analyze the expression and size of the fusion protein (Fig 3B). Transgenes in *Chlamydomonas* integrate at random locations, which could affect expression efficiency. In addition, the transgene plasmid is often broken or rearranged in *Chlamydomonas* before it is integrated. The Western blot analysis and genetic rescue ensures an approximately full-length fusion protein, but in principle the promoter region could be disrupted causing less efficient transcription. Perhaps for these reasons, expression levels vary among transformants. For additional experiments we chose clones with efficient rescue and protein expression. These clones were backcrossed with wild type at least once before use in further experiments (Fig 3, red numbers).

## Expression of DNA replication proteins during the division cycle

**Fig 4:**
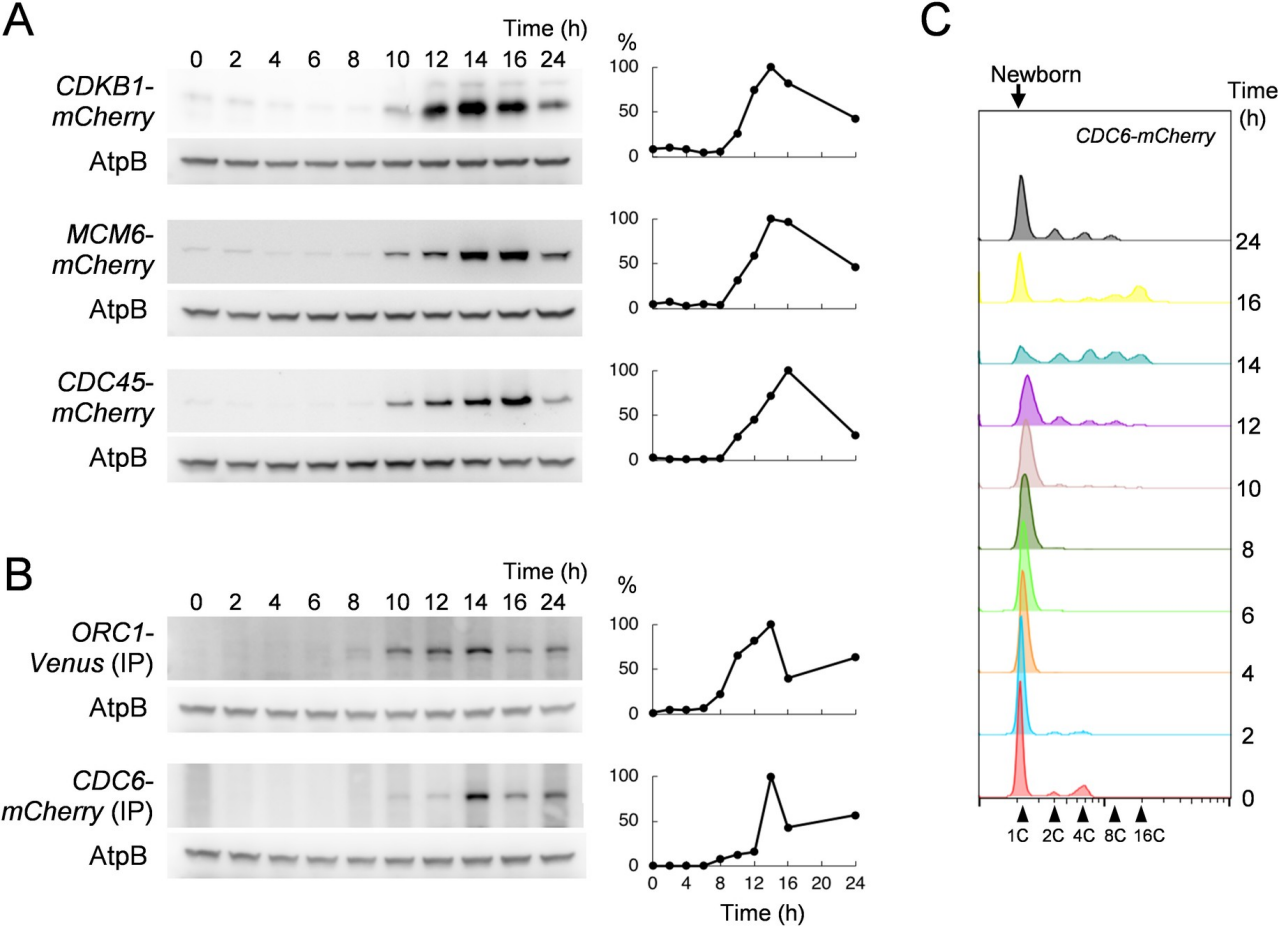
DNA replication proteins are expressed during cell division. (A) *CDKB1-mCherry*, *MCM6-mCherry* or *CDC45-mCherry* cells were synchronized in G1 by nitrogen starvation. The cell cycle was released and cells were collected at indicated time points. Protein was extracted and subjected to western blotting analysis. The signal was detected by anti-mCherry antibody. AtpB was used as a loading control. The relative protein expression quantified by ImageJ is shown on the right. The highest expression during the cell cycle was set as 100%. (B) *ORC1-Venus* or *CDC6-mCherry* cells were prepared as described in A. Protein was pulled down by GFP or mCherry nanobody coupled with DynaBeads. The signal was detected and analyzed as described above. (C) The *CDC6-mCherry* samples used in B were fixed, stained and subjected to FACS analysis to monitor cell cycle progression.

All transgenes were constructed with their native promoters, so that we could analyze accumulation of MCM6mCherry, CDC45-mCherry, ORC1-Venus and CDC6-mCherry fusion proteins at approximately endogenous levels throughout the cell cycle. We included a strain expressing CDKB1-mCherry as a positive control since CDKB accumulates specifically in division-phase cells [40]. Cells were arrested at G1 phase as described above, then released into complete media at 33°C. Since the transgene was expressed in the context of the endogenous ts-lethal mutation, cell cycle progression was dependent on the fluorescent fusion protein in each case. CDKB1-mCherry was first expressed at 10 hrs, peaked around 14 hrs and declined sharply by 24 hrs as previously shown (Fig 4A) [40]. The time of high CDKB expression coincides with the period of multiple fission cycles. Similarly, the maximum levels of tagged MCM6, ORC1, CDC6 and CDC45 were observed at 14 hrs after release which is consistent with published transcription profiles (Fig 4A and 4B) [6]. ORC1 and CDC6 mCherry fusion protein levels were undetectable by direct Western analysis. Therefore, these tagged proteins were pulled down using Dynabeads conjugated with mCherry nanobody to concentrate the protein before Western blot analysis. FACS analysis of *CDC6-mCherry* cultures showed that DNA replication initiated at 12 hrs as expected in this procedure (Fie 4C). We conclude that pre-RC proteins are essentially absent during the long G1 phase in *Chlamydomonas*, and like CDKB1, are expressed only during the rapid divisions of the multiple fission cycle. This is an interesting contrast to the yeast system, in which pre-RC components are loaded onto origins starting in late mitosis and are bound throughout G1 phase. MCM4 expression at a single cell level was further analyzed by time lapse fluorescent microscopy (see below).

### MCM proteins are localized to the nucleus during the division cycle with a transient release during mitosis

**Fig 5:**
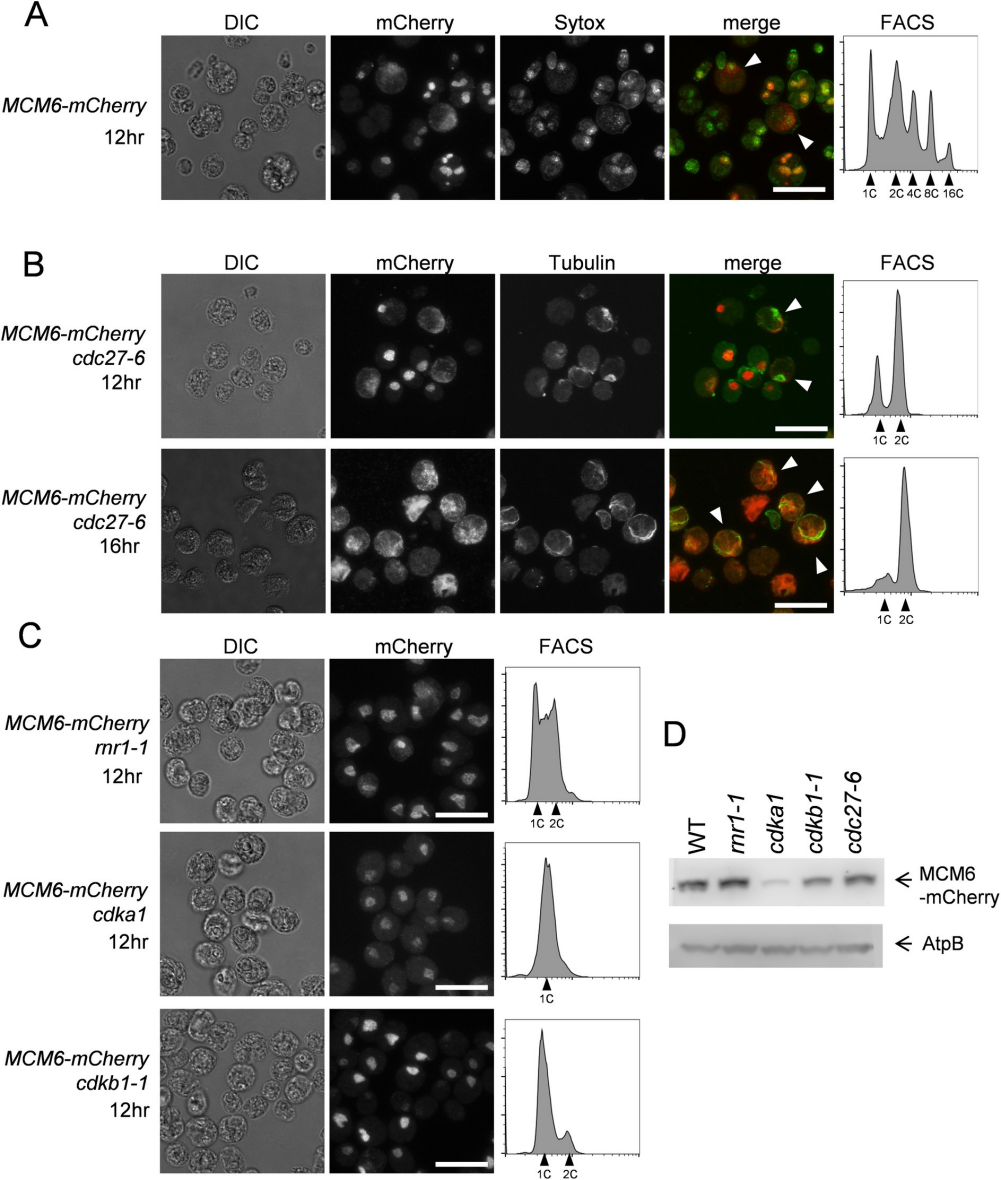
MCM6 is localized to the nucleus. (A) *MCM6-mCherry* cells (WT) were arrested in G1 and released at 33°C for 12 hrs. Cells were fixed and stained with anti-mCherry antibody (Red) and Sytox green (Green). White arrows show cells with diffused MCM6. Images were acquired by Confocal microscope. Scale bars = 20 μm. The DNA content was analyzed by FACS (right panel) (B) *MCM6-mCherry cdc27* cells were collected after 12 or 16 hrs from release and prepared as described in A. White arrows show cells with mitotic spindles. (C) *MCM6-mCherry rnr1*, *MCM6-mCherry cdka1* or *MCM6-mCherry cdkb1-1* cells were collected after 12 hrs from release. Cells were prepared as described in A. (D) Indicated strains were arrested in G1 and collected 12 hrs after release. Protein was extracted and MCM6 was detected by anti-mCherry antibody. AtpB was used as a loading control.

MCM6-mCherry protein localization was analyzed by indirect-immunofluorescence using anti-mCherry antibody. MCM6 was localized to the nucleus in wild type cells, but we detected sporadic cells with dispersed MCM6 protein in the cytoplasm (Fig 5A, white triangles). We used a *cdc27-6* cell cycle mutant in order to examine MCM6 localization in metaphase*-*blocked cells. CDC27 is a part of the Anaphase Promoting Complex (APC) [41] required for the metaphase-anaphase transition [35,40]. MCM6 was diffused from the nucleus in metaphase-arrested *cdc27-6* cells, and co-staining of microtubules showed that these cells contained mitotic spindles (Fig 5B). After 16 hrs the cell cycle was completely arrested in G2/M phase after completion of a single round of DNA replication in the *cdc27-6* mutant (Fig 5B). Most likely, the sporadic wild type cells with diffuse MCM6-mCherry localization are cells transiting through metaphase.

In budding yeast, translocation of Mcm2-7 to the cytoplasm after origin firing may prevent origin re-loading within a given S-phase [42]. Localization of Mcm2-7 is controlled by Cdk1-dependent phosphorylation [18,43]. For example, Mcm2 and Mcm3 contain nuclear localization signals (NLS). Mcm3 undergoes CDK-dependent phosphorylation at an NLS-NES module to promote protein transport of the whole Mcm2-7 complex [18]. It is possible that transient mitotic loss of MCM6 from the nucleus in *Chlamydomonas* is regulated similarly. We tested if CDKA1 or CDKB1 controls MCM6 localization and found that MCM6 stayed in the nucleus in both *cdka1* and *cdkb1-1* mutants (Fig 5C), while leaving the nucleus in the *cdc27-6* background in which CDKA1 and CDKB1 activities are both high [40]. Thus, it is possible that CDK activity regulates MCM6 localization in *Chlamydomonas* as in yeast. Western blotting analysis showed that the MCM6-mCherry protein was readily detectable in *rnr1-1*, *cdkb1-1* and *cdc27-6* mutants similar to the level in wild type cells and was slightly suppressed in the *cdka1* mutant (Fig 5D). RNR1 is a major form of the large subunit of ribonucleotide-diphosphate reductase, which catalyzes dNTP synthesis. The *rnr1-1* mutant arrests the cell cycle before the first DNA replication (Fig 1), but presumably does not prevent pre-replicative complex formation.

**Fig 6:**
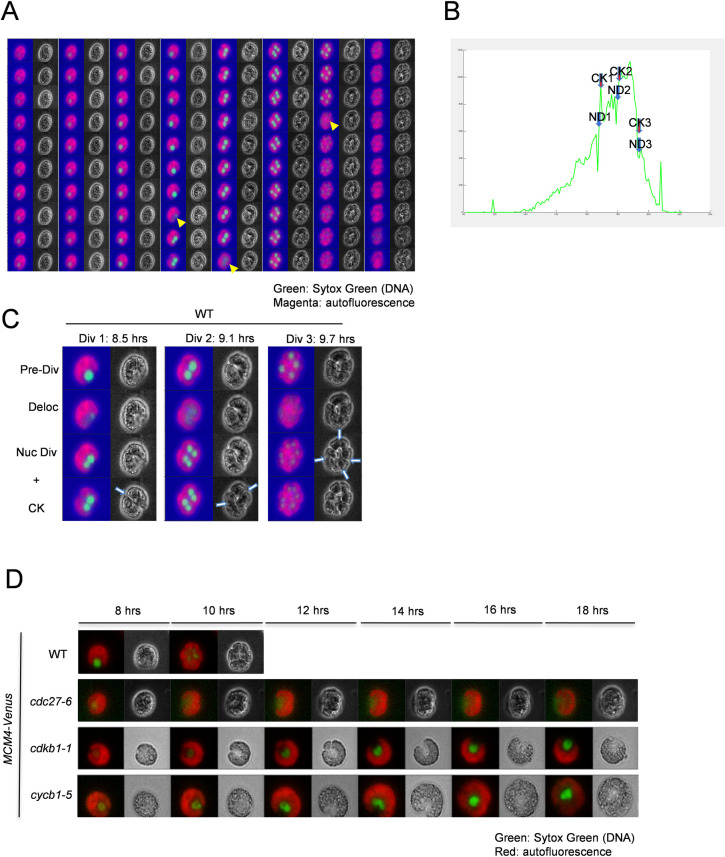
MCM4 is briefly translocated to the cytoplasm in every single S/M phase. (A) *MCM4-Venus* cells were removed from nitrogen starvation and placed on TAP for live imaging by time-lapse microscopy. MCM4-Venus (Green) and chloroplast auto-fluorescence (Magenta). Yellow arrows indicate the time when MCM4-Venus disappeared from the nucleus. (B) The relative intensity of MCM4-Venus signal across the cell in A was quantified. ND: nuclear division, CK: cytokinesis. (C) The representative images of four stages (Pre-Div: pre division, Deloc: delocalization, Nuc Div: nuclear division, CK: cytokinesis) are shown for 1st, 2nd and 3rd division in wild type, Blue arrows indicate the division site. (D) Live cell time-lapse microscopy of *MCM4-Venus* in WT, *cdc27-6*, *cdkb1-1*, and *cycb1-5* backgrounds.

To extend these findings to a different member of the MCM complex, we monitored MCM4-Venus localization in single cells, using a variant of procedures developed for budding yeast time-lapse microscopy [44,45]. Cells expressing MCM4-Venus were monitored at 3 min resolution, starting in G1 and extending to completion of a multiple fission cycle. MCM4-Venus was absent in the initially plated G1 cells but began to accumulate approximately 2 hrs before the first division (Fig 6A and S1 Movie). MCM4-Venus was localized to a small region in the cell anterior that we assume is the cell nucleus based on size, shape, position and similarity to the MCM6-mCherry nuclear localization in immunofluorescence experiments in Fig 5. The MCM4-Venus signal was greatly reduced from the nucleus in the frame right before the first division but reappeared almost immediately after division (Fig 6A and 6C). Quantification of the total MCM4-Venus signal across the cell indicated that the reduction or loss of nuclear intensity occurred without significant change in total cellular levels (Fig 6B). Therefore, we attribute the loss of nuclear signal to transient diffusion into the cytoplasm rather than to MCM4 proteolysis and rapid resynthesis. MCM4-Venus transient loss was restricted to a single 3-min frame in almost all cells observed. We observed the same one-frame loss of MCM4-Venus nuclear intensity right before the second and third divisions, again with no notable change in total cellular MCM4-Venus levels (Fig 6A and 6C). As with the first division, MCM4-Venus nuclear signal returned immediately upon cytokinesis at completion of the later divisions. In the *cdc27-6* background, MCM4-Venus was localized in the nucleus in G1 but delocalized as cells entered the *cdc27-6* arrest. In *cdc27-*arrested cells, MCM4-Venus delocalization was stable for many hours (Fig 6D and S2 Movie). This contrasts with wild type, where MCM4-Venus delocalization lasted for only ~3 min coincident with cell division (Fig 6C). MCM4-Venus diffusion from the nucleus was dependent on CDKB1, since MCM4-Venus remained stably nuclear throughout an 18 hour timelapse movie of *cdkb1-1* cells (Fig 6D and S3 Movie). The *cycb1-5* mutant also showed sustained MCM4-Venus nuclear localization similar to that in the *cdkb1-1*mutant (Fig 6D and S4 Movie). Since spindle formation is dependent on CDKB1 and CYCB1, these results indicate a metaphase dispersal of MCM4, just as was observed with MCM6 above, and suggest that the entire MCM complex may transiently delocalize from the nucleus to the cytoplasm at metaphase.

**Fig 7:**
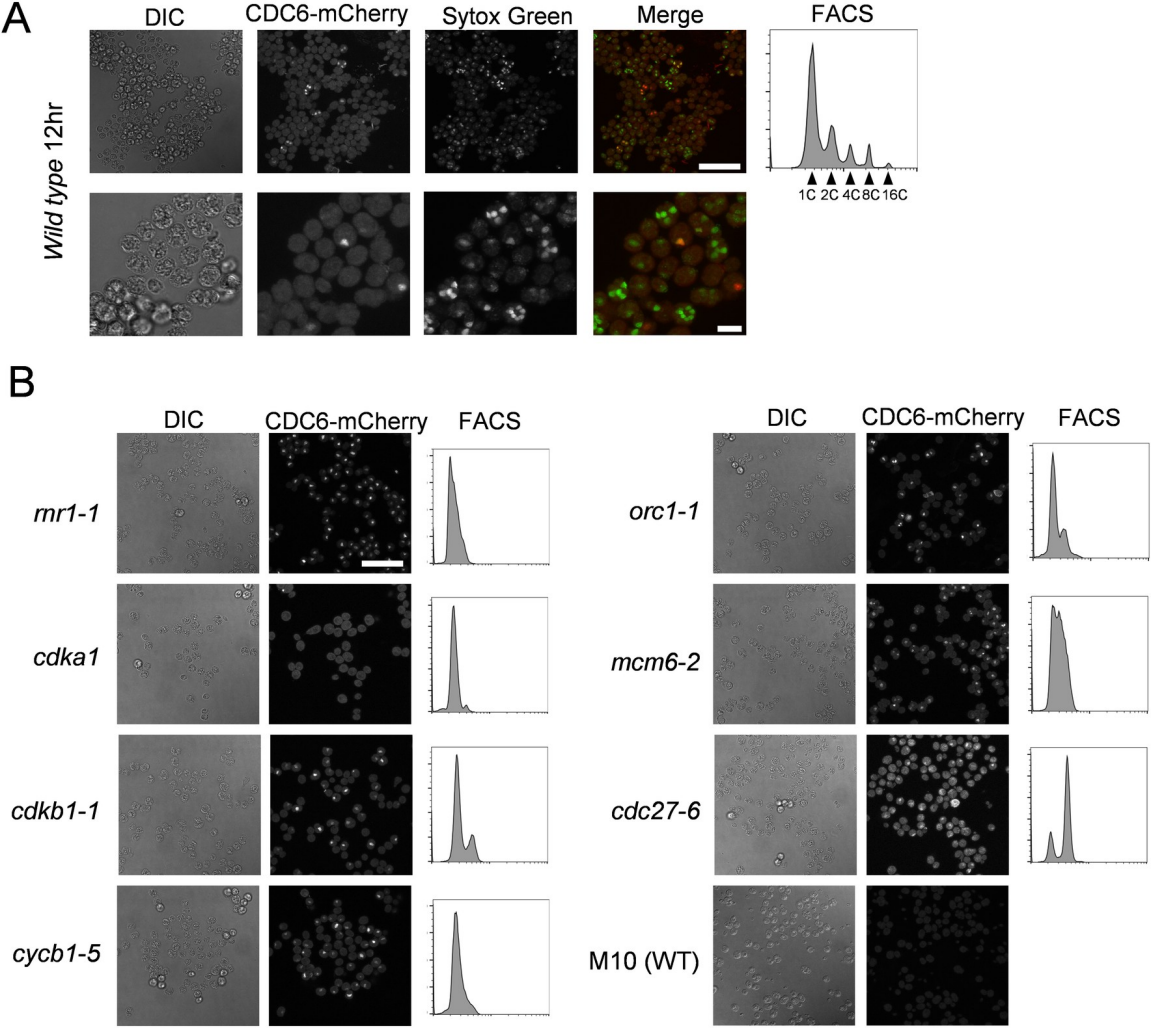
CDC6 protein is transiently localized to the nucleus in dividing cells. (A) *CDC6-mCherry* (Wild Type) cells were synchronized in G1 and collected 12 hrs after release. Cell were fixed and stained with anti-mCherry antibody (Red) and Sytox green (Green). Images were acquired by Confocal microscope. Scale bar = 50 μm (upper row) and 10 μm (lower row). DNA content was analyzed by FACS (right panel). (B) *CDC6-mCherry* in *rnr1-1*, *cdka1*, *cdkb1-1*, *cycb1-5*, *orc1-1*, *mcm6-2* or *cdc27-6* mutants were synchronized in G1 and collected 14 hrs after release. Cell were fixed and stained with anti-mCherry antibody. Images were acquired by Confocal microscope. DNA content was analyzed by FACS (right panel). M10 strain without *CDC6-mCherry* was used as a negative control to show background signal. Scale bar = 50 μm.

To determine if this transient protein delocalization in metaphase is specific to the MCM proteins, we used a strain expressing a bleomycin-resistance gene fused to GFP (Ble-GFP). Unlike free GFP, Ble-GFP is nuclear-localized [46]. Much as with MCM4-Venus, Ble-GFP nuclear signal was sharply reduced for about 3 min right at the time of cell division, with no decrease in total cellular Ble-GFP signal (S5 Movie). Ble-GFP in the *cdc27-6* background diffused from the nucleus as cells entered a *cdc27-6* arrest and failed to re-concentrate for the duration of the movie (S6 Movie). Thus, Ble-GFP reconstitutes the cell-cycle-regulated nuclear-cytoplasmic traffic observed with MCM4 and MCM6. We were unable to analyze ORC1-Venus and CDC6-Venus by time-lapse microscopy, presumably due to their low abundance as noted above from Western analysis in Fig 4B. However, cytoplasmic diffusion of CDC6-mCherry was observed by indirect immunofluorescence in the *cdc27-6* mutant, in contrast to the nuclear concentration observed in wild type (Fig 7B; see below).

### CDC6 protein levels are dependent on CDKA1 activity

By immunofluorescence, CDC6-mCherry was detectable in cell nuclei, although almost never in cells with more than two nuclei. These cells, in general, underwent 3–4 division cycles (yielding 8–16 nuclei) (Fig 7A), suggesting that the later cycles occurred with very low (undetectable) levels of CDC6. The model from budding yeast (see Introduction) suggests that CDC6 should be required at the end of each mitosis to license replication for the succeeding rounds. We don’t know if this is a real difference between the systems or if late rounds of replication in *Chlamydomonas* can be licensed with very low levels of Cdc6.

In budding yeast, Cdc6 protein is regulated by transcription, localization and protein degradation (see Introduction). Cdc6 is phosphorylated by Cdk1 and targeted for ubiquitination followed by proteasome-mediated protein degradation [14–16,47–49]. Therefore, we tested if *Chlamydomonas* CDC6-mCherry protein levels are affected by inactivation of *CDKA1* or *CDKB1*. CDC6-mCherry was transiently localized to the nucleus during the division cycle in wild type cells and also nuclear-localized in *rnr1-1*, *cdkb1-1*, *cycb1-5*, *orc1-1* and *mcm6-2* mutants (Fig 7A and 7B). By contrast, in the *cdka1*::HYGRO disruption mutant, CDC6-mCherry was completely undetectable (Fig 7B). Thus, CDKA1 is required for efficient CDC6 accumulation. In yeast, CDK activity results in destabilization and lower CDC6 levels, while in humans, CDK-dependent HsCdc6 phosphorylation prevents Cdc6 proteolysis [24,25]. *Chlamydomonas* may resemble humans more than yeast in this respect.

**Fig 8:**
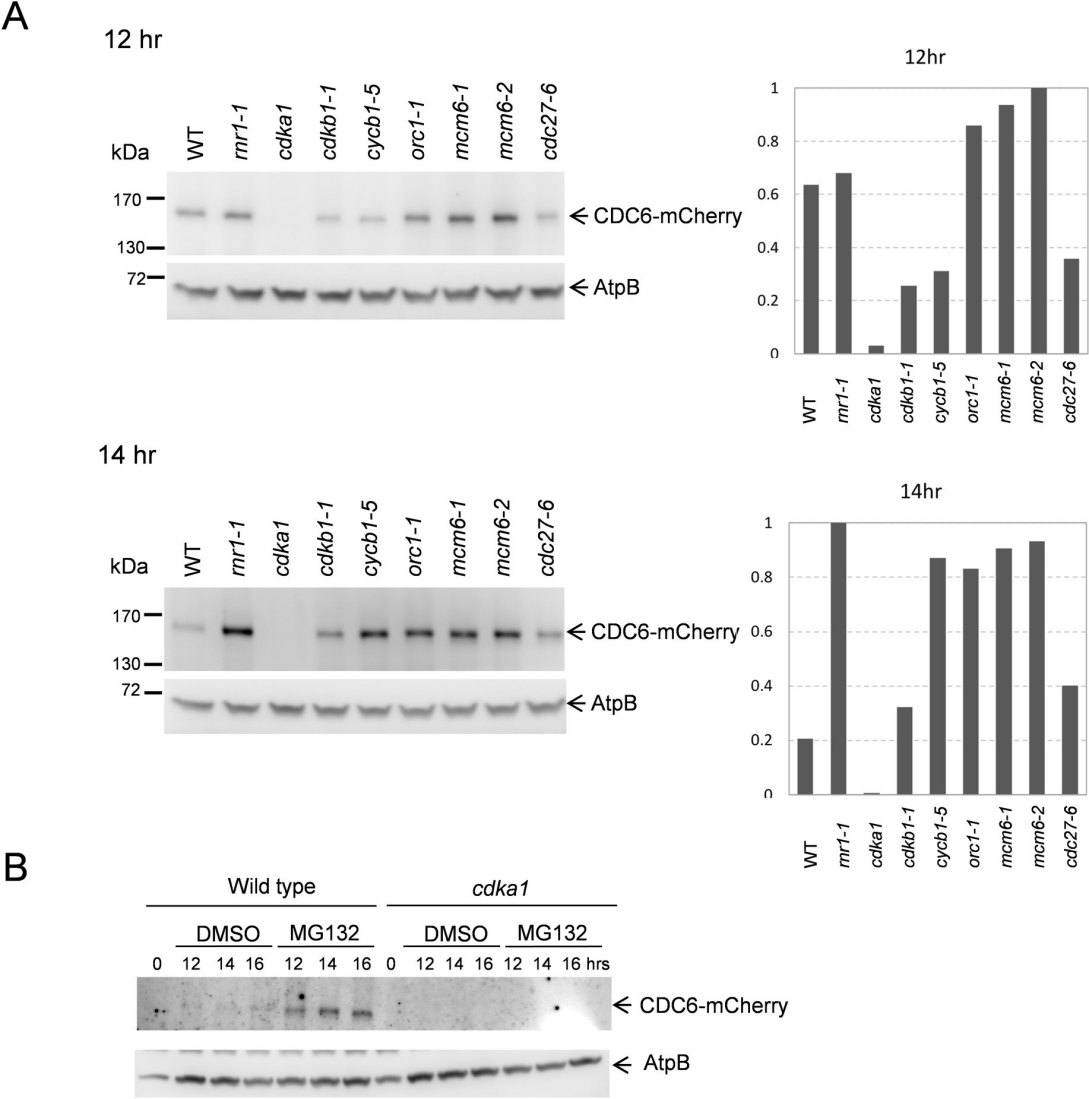
CDC6 protein expression in cell cycle mutants (A) Indicated strains were arrested in G1 and collected 12 or 14 hrs after release. Protein was extracted and CDC6-mCherry was pulled down using mCherry nanobody coupled with DynaBeads. The signal was detected by anti-mCherry antibody. AtpB was used as a loading control. The graph shows quantification of relative expression in each strain. (B) *CDC6-mCherry* (WT) or *CDC6-mCherry cdka1* (*cdka1*) cells were arrested in G1 and plated on TAP plates to release the cell cycle arrest. After 10 hrs from release, cells were plated on TAP with DMSO or MG132. Cells were collected 12, 14 or 16 hrs after release corresponding to 2, 4 or 6 hrs treatment. Proteins were extracted and CDC6-mCherry was pulled down using mCherry nanobody coupled with DynaBeads. The signal was detected by anti-mCherry antibody. AtpB was used as a loading control.

To confirm and extend these findings, we examined CDC6-mCherry protein level by western blot in various mutant backgrounds. Results were consistent with the immunofluorescence results: CDC6-mCherry was observed in *rnr1-1*, *cdkb1-1*, *cycb1-5*, *orc1-1*, *mcm6-1*, *mcm6-2* and *cdc27*-6 after 12 hrs from release (Fig 8A), but was undetectable in the *cdka1*::*HYGRO* mutant at 12 and 14 hrs after release (Fig 8A). This finding was reproduced with three independent *CDC6-mCherry cdka1* clones, where in all cases the *CDC6-mCherry* transgene efficiently rescued ts–lethality of the *cdc6-1* mutation. *CDC6* is still essential in a *cdka1* background, since *cdka1;cdc6-1* double mutants are temperature-sensitive but *cdka1;CDC6* single mutants are not. This finding implies that there must be an undetectable but still functional level of CDC6 expressed in the *cdka1* mutant. The prolonged delay in replication in *cdka1* mutants [5] may be due at least in part to very low levels of this essential replication protein.

### *Chlamydomonas* CDC6 is degraded by the proteasome

In yeast, CDK-phosphorylated Cdc6 is degraded by ubiquitin-mediated proteolysis [14–16,47,49]. We examined *Chlamydomonas* CDC6 accumulation in the presence of a proteasome inhibitor, MG132. Cells were released from G1 and collected after 12, 14 or 16 hrs. In wild type cells, CDC6 levels were increased by MG132 treatment (Fig 8B), suggesting that CDC6 is degraded by the proteasome. However, MG132 treatment did not result in detectable CDC6 accumulation in the *cdka1* background (Fig 8B). Therefore, CDC6 suppression in the *cdka1* mutant is unlikely to be due solely to excessive proteasomal degradation. In animal cells, MG132 blocks cells in metaphase. However, in our experiments, the *Chlamydomonas* cell cycle profile was not significantly changed by treatment. We do not know if failure of MG132 to arrest the *Chlamydomonas* cell cycle is due to inefficient proteasome inhibition at the levels we used or for some other reason. However, the cell cycle progression in MG132-treated cells does allow us to conclude that the CDC6 stabilization we observe is not due to an indirect effect of cell cycle arrest.

### MCM4 was tightly bound on chromatin in *rnr1* and *cdkb1*, but not in *orc1*, *cdc6* and *mcm6* mutants

**Fig 9:**
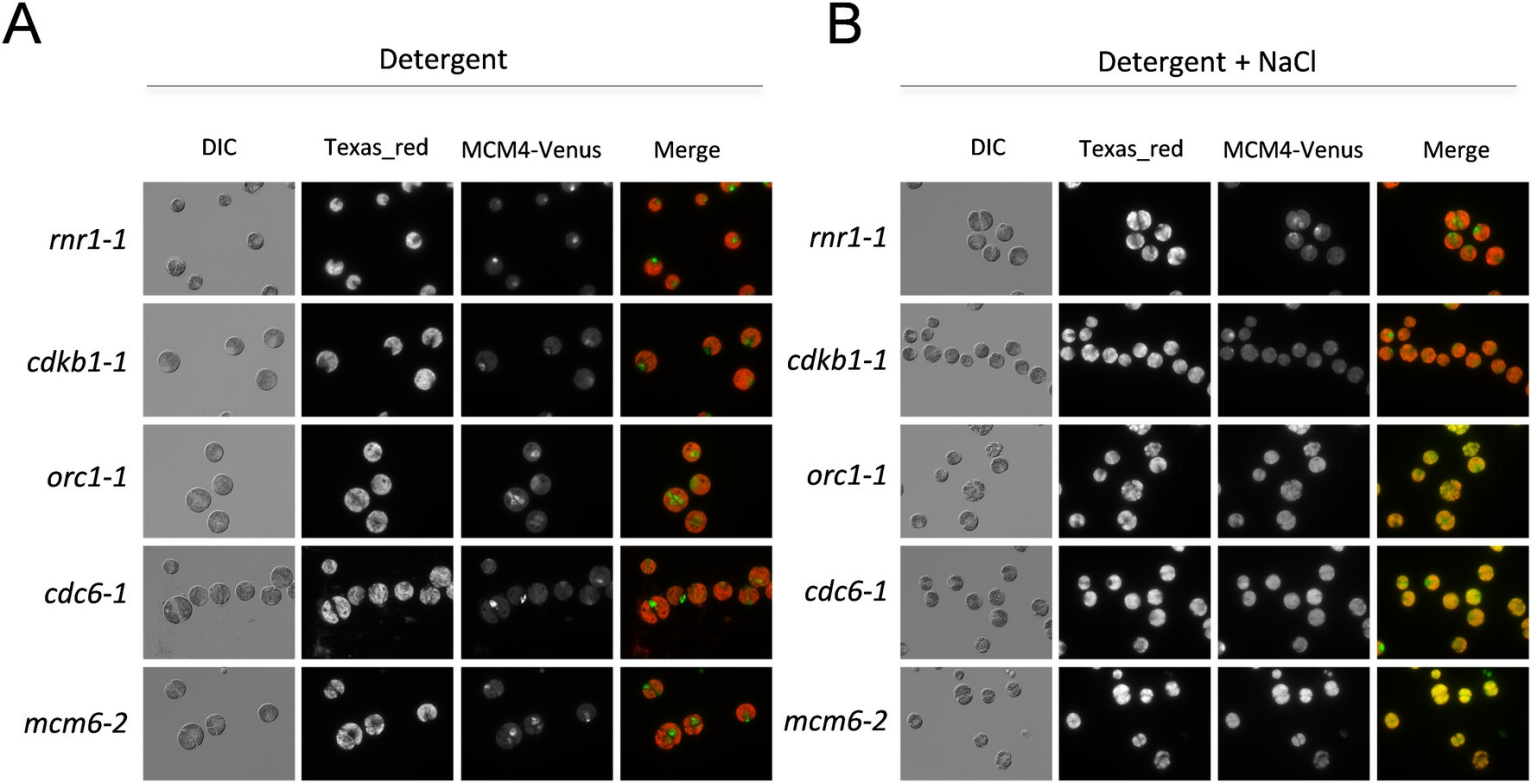
Nuclear MCM4 is salt-resistant in *rnr1* and *cdkb*, but not in *orc1*, *cdc6* and *mcm6* mutants. (A) *MCM4-Venus in rnr1-1*, *cdkb1-1*, *orc1-1*, *cdc6-1 or mcm6-2* were arrested in G1 and released at 33°C for 12 hrs. Cells were treated with both 0.03% TritonX and 0.03% Digitonin together to permeabilize membranes. MCM4-Venus was observed under a fluorescence microscope (Green). Texas Red shows auto fluorescence (Red). (B) Cells were further treated with 0.25M NaCl to release chromatin bound MCM4 from the nucleus.

MCM helicase is bound to DNA dependent on ORC complex and CDC6 in opisthokonts, forming pre-RC. Cdk1 activity further transforms the pre-RC into pre-initiation complex (pre-IC) containing active helicase where MCM2-7 hexamer is tightly loaded on chromatin [11,50]. The status of MCM2-7 loading has been studied by a stepwise pre-RC assembly assay *in vitro* using purified yeast proteins [12,51]. In these assays, MCM loading on chromatin is initially sensitive to high concentrations of salt, but transits to a salt-resistant complex upon full loading. We do not have recombinant *Chlamydomonas* proteins to carry out similar experiments, but we reasoned that we could monitor MCM4-Venus nuclear localization in detergent-permeabilized cells in low or high salt concentrations. First, *Chlamydomonas* plasma membranes in division cycle were permeabilized with Triton X-100 and Digitonin. In animal cells, nuclear envelopes remain structurally intact after such a detergent treatment [52]. Treatment with 0.03% Triton-X and 0.03% Digitonin allowed DNA staining with ethidium bromide, which was not achieved without detergent treatment (S3B and S3C Fig), indicating effective permeabilization of the plasma membrane. Nuclear ethidium bromide staining was resistant to salt treatment with 0.25M NaCl, confirming that nuclei were not globally disrupted by high salt (S3D Fig). Next, we tested if MCM4-Venus stays in the nucleus in *rnr1-1*, *cdkb1-1*, *orc1-1*, *cdc6-1* and *mcm6-2* mutants in permeabilized cells, with or without high-salt treatment. MCM4-Venus was in the nucleus before and after detergent treatment in all mutant backgrounds (Fig 9A). However, in the *orc1-1*, *cdc6-1* and *mcm6-2* backgrounds, MCM4-Venus localization became diffuse through the cell upon treatment with high salt. This finding correlates with the requirement for ORC, CDC6 and the other MCM proteins for salt-resistant binding of the MCM complex in the *in vitro* yeast experiments discussed above [12]. MCM4-Venus stayed in the nucleus in *rnr1-1* and *cdkb1-1* mutants even after salt treatment suggesting that RNR1 and CDKB1 were dispensable for MCM4 chromatin loading (Fig 9B).

## Discussion

### A conserved set of replication control proteins in the plant kingdom

Our high-throughput hunt for temperature-sensitive mutants generated hundreds of cell cycle mutants, for which the causative mutations were determined by multiplexed bulked-segregant sequence analysis [35,36]. A number of these mutations fell in proteins aligning with replication control proteins defined in other eukaryotes. We show here that these mutants were indeed defective in DNA replication, indicating that their core functions as well as their sequences are conserved (Fig 1). We also noted that *cdc6* mutant showed a wide peak between 1-2C DNA content while other replication mutants showed a tight 1C arrest. The *cdc6* mutant might be less tightly temperature sensitive compared to others; we have only a single allele so this is hard to evaluate.

Inhibition of replication by any of these mutants also blocked mitotic spindle formation (Fig 1C). This could reflect a checkpoint preventing mitotic entry until completion of replication as observed in many other eukaryotes [38]. A checkpoint of this kind may thus be conserved in *Chlamydomonas* and probably in the plant kingdom overall [53]. In contrast, another well-known checkpoint mechanism, the spindle assembly checkpoint which blocks mitotic exit and the next round of DNA replication upon spindle disruption, is hard to detect in *Chlamydomonas* [35], causing at most a transient block to multiple cycles of replication.

### A loss of nuclear retention in a “closed mitosis”

Whether the nuclear envelope breaks down in mitosis or remains intact (‘open’ vs. ‘closed’ mitosis) is a significant phylogenetic marker, with multiple independent transitions in different lineages [54]. For example, *S*. *cerevisiae* and *S*. *pombe* have closed mitosis while *S*. *japonicas* has semi-open mitosis due to extended spindles which causes nuclear envelope tearing [55]. *U*. *maydis* exhibits open mitosis with nuclear envelope rupture [56]. The fungus *A*. *nidulans* shows partial nuclear pore complex disassembly in closed mitosis [57]. Most higher eukaryotes have open mitosis with a complete nuclear membrane breakdown. *Chlamydomonas* is traditionally classified as having a closed mitosis; however, permeability of the nuclear envelope in a closed mitosis may vary among organisms [58]. In *Chlamydomonas*, MCM4 and MCM6 were localized to the nucleus throughout the division cycle except during mitosis, when they transiently diffused to the cytoplasm; CDC6 showed a similar localization pattern during mitosis (Figs 5–7). Transient MCM protein diffusion during mitosis is also observed in *Arabidopsis* [30], which has an open mitosis.

**Fig 10:**
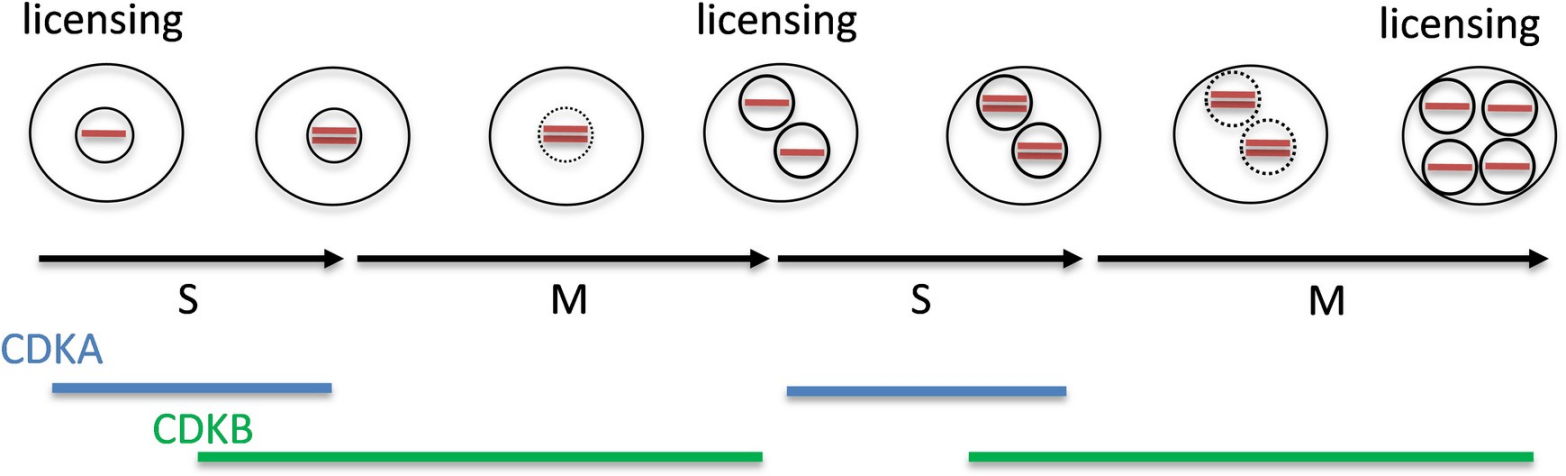
A model of origin licensing in *Chlamydomonas*. Red bar shows DNA.

*Chlamydomonas* spindle poles are outside the nuclear envelope and the spindle enters the nucleus through large holes called polar fenestrae [59]. The fenestrae are 300-500um in diameter, 5 times larger than nuclear pores in interphase. If the fenestrae are truly open channels, this should allow rapid diffusion of all nuclear components that are not tightly bound to large structures (such as chromosomes, mitotic spindles or a nuclear matrix). We observed ble-GFP transient diffusion in mitosis which supports the idea that the protein diffusion is not specific to DNA replication proteins, but rather due to a general loss of nuclear integrity (S5 and S6 Movies). This transient protein diffusion might therefore be functionally equivalent to nuclear membrane breakdown in organisms showing ‘open mitosis’. Nuclear envelope breakdown is triggered in early mitosis and nuclei are reassembled at the end of mitosis. In an early proposal to explain ‘licensing’ of replication origins in *Xenopus*, it was proposed that the nuclear envelope excludes access of key replication factors to fired origins; nuclear envelope breakdown allows access and subsequent origin licensing [60]. We speculate that the fenestrae in *Chamydomonas* may serve as a related cell cycle reset mechanism to license origins, which might be necessary for the unique and rapid division cycles with repeated S/M phases without distinct G1 phase once cells enter division cycle (Fig 10). Our results suggest that the classic distinction between ‘open’ and ‘closed’ mitosis may not imply restricted traffic of nuclear components [58].

### Pre-RC formation in *Chlamydomonas*

All of the DNA replication proteins we analyzed were essentially absent during the long G1 before multiple fission (Fig 4). This correlates with transcriptional profiles of replication genes [5,6]. In yeast, CDK activity is very low specifically in late mitosis and early G1, and low CDK activity is permissive for pre-RC formation and origin loading with the MCM helicase; loaded origins persist through G1 until firing occurs under positive CDK control. The essential absence of pre-RC proteins in *Chlamydomonas* during G1, despite very low CDK activity, suggests that origin loading may be regulated differently than in yeast, since during most of G1 there appears to be negligible levels of pre-RC proteins. The origin licensing may happen right before S-phase after the protein diffusion (Fig 10).

We further showed that MCM2-7 loading is dependent on ORC and CDC6, suggesting that the mechanism on pre-RC assembly is conserved across all of the eukaryotes (Fig 9). Furthermore, CDKB1 was dispensable for MCM2-7 loading on the chromatin. It is very likely that CDKA1 and CDKB1 overlap in controlling initiation of replication [35,40]; formally similar to the multiple B-type cyclins in yeast (Fig 10)[61].

### Inhibition of DNA re-replication

In yeast, CDK phosphorylates the MCM complex at NLS-NES modules, which triggers its nuclear export during S-phase after individual origins have fired [18]. In contrast, *Chlamydomonas* MCM4 stays in the nucleus throughout the cell cycle except for a window of ~3 min, indicating that CDK probably does not exclude MCM4 from the nucleus during S-phase. *Chlamydomonas* MCM2 and MCM6 contain candidate NLS motifs, however there are no CDK sites nearby (S4 Fig). It suggests that MCM2-7 protein does not use this system. It is not clear how reloading of MCM2-7 complex is inhibited after initiation. One possibility is that CDKB1 dependent MCM phosphorylation may inhibits the function in the nucleus.

CDC6 stayed in the nucleus in the *cdkb1* mutant (Fig 7). However, CDC6 was undetectable in the *cdka1* mutant (Figs 7 and 8). This was a surprising result because Cdc6 degradation is mediated through CDK-dependent phosphorylation in yeast. *CDKA1* transcription peaks earlier than *CDKB1* [5,6], and efficient and timely expression of *CDKB1* and DNA replication genes depends on CDKA1 [5]. Thus, the CDC6 suppression in the *cdka1* mutant might be a reflection of this transcriptional inhibition. It is known that Cdc6 regulation is diverse across the species. In budding yeast, CDK mediates SCF-dependent Cdc6 degradation to inhibit pre-RC formation in S-phase. In humans, APC-dependent Cdc6 proteolysis inhibits Cdc6 accumulation, and CDK phosphorylates and protects Cdc6 to trigger origin licensing [24]. *Chlamydomonas* CDC6 contains three conserved CDK consensus site, S/T-P-X-K/R with one phospho-degron motif at amino acid 214–221 (S4 Fig). CDC6 level was not elevated in the *cdkb1* mutant, therefore it is unclear whether any of these mechanisms will apply in *Chlamydomonas*. Proteasome-dependent degradation of CDC6 may provide a first clue to Cdc6 regulation (Fig 8B).

*Chlamydomonas* ts-lethal mutations in many proteins controlling DNA replication allow a detailed functional examination of DNA replication control in the plant kingdom. Absence of an accessible culture system or conditional alleles, and a high level of gene duplication makes such experimentation much more difficult in land plants. Thus, *Chlamydomonas* is a valuable model for replication control in the critically important plant kingdom.

The budding yeast DNA replication control system has been characterized in depth and detail, providing specific molecular hypotheses to evaluate in *Chlamydomonas*. The results reported here already demonstrate conserved features but also some likely and significant differences. It is important to note that conservation of sequence alone does not guarantee conservation of function or regulation. Conditional mutants are key resources for functional analysis.

## Materials and methods

### Cell culture conditions

All strains are listed in S1 Table. Cells were maintained on Tris-acetate-phosphate (TAP) medium (Harris, 2008) at 21°C under continuous illumination at ~100 PAR (determined with Apogee Quantum Light Meter). In order to synchronize cells in G1 by nitrogen depletion, cells were spread on 0.1xN TAP plates (containing 1/10 of standard TAP levels of ammonium chloride) and incubated for 2 days at 21°C under the light. The G1 arrest was released by plating cells on TAP plates at 33°C under continuous illumination at ~150 PAR.

### Strain cross and mutant analysis

Strain cross was performed as described previously [62]. Strain list is in S1 Table. Tetrad haploid progenies were dissected with a Zeiss Axioskop 40 Tetrad microscope. Mutations were verified by allele-specific competitive PCR (Onishi M). Briefly, three primers were designed for each mutant. Two forward primers were designed to distinguish wild type and mutant by a length of PCR product. Forward primer 1 contains wild type sequence with 20bp long. Forward primer 2 contains the mutant sequence with 40bp long. A reverse primer was designed to recognize sequence a few hundred base pairs downstream of the mutant site. PCR reaction was performed using these 3 primers, and the PCR product was analyzed using 3% SB agarose gel. The PCR band with the mutation is 20bp longer than that with wild type. Primer sequences are listed in S2 Table.

### Plasmid construction and transformation

The plasmid backbone was generated from *CDKB1* plasmid which contains mCherry-tagged *CDKB1* gene [40]. The mCherry plasmid *FC1011* plasmid was constructed by NEBuilder HiFi DNA assembly Master Mix (New England Biolabs, MA) using pKA1 (*CDKB1* plasmid) as a temperate [40]. The mCherry fragment was replaced by GFP and Venus to create GFP- and Venus-universal plasmids, named *FC1012* and *FC1001* using multiple cloning sites. The desired gene (*MCM6*, *CDC45*, or *CDC6*) was amplified from genomic DNA and inserted into the mCherry plasmid at the multiple cloning site. *ORC1-mCherry* and *MCM4-VENUS* plasmids were synthesized by SynBio (NJ). For these plasmids, all of the introns except the first two and the last intron were removed. The plasmid linearized by XbaI digestion was used for transformation.

The final plasmids contain *aphVIII CDS* for paromomycin resistance that is useful for drug selection in *Chlamydomonas*, an Ampicillin resistant gene for bacterial selection, and mCherry, GFP or Venus followed by the *CDKB1* 3’UTR, as well as multiple cloning sites for insertions of desired promoters and coding sequences (S1 Fig).

Temperature sensitive mutant cells were transformed with the linearized plasmid by electroporation (500 V, 50 μF, 800 Ω) using GenePulser Xcell (Bio-Rad, CA). The cells were recovered in TAP containing 40 mM sucrose, incubated under light overnight, and then plated on a TAP plate containing 10 ug/ml paromomycin. Complementation of temperature sensitivity among paromomycin-resistant transformants was determined by serial dilution of cells on TAP plate at 33°C for 2–5 days. Plasmid integration and structure was confirmed by PCR.

We used *mCherry* tagged strains for indirect immunofluorescence because anti-mCherry antibody generally works better than anti-GFP antibody. For time-lapse microscopy, mCherry signal is hard to detect due to chloroplast autofluorescence in *Chlamydomonas*, but cell-cycle-regulated accumulation and localization of MCM4-Venus was clearly detectable at high time resolution (Fig 6).

### Flow cytometry

Flow Cytometry analysis (FACS) was performed as described [35] using a BD Accuri C6 instrument (BD Biosciences). The data was analyzed by FlowJo software (FlowJo, LLC, OR).

### Western blotting

Cells were lysed in RIPA buffer containing protease inhibitors with glass beads using FastPrep (MP Biomedicals, CA) for 20s, twice, at speed 6. Cleared cell lysate was denatured with SDS buffer and separated by SDS–PAGE with Novex 4–12% Tris-glycine polyacrylamide gel (Invitrogen, Life Technologies, CA). Western blot analysis was performed using anti-mCherry antibody (16D7) at 1:2000 dilution (Thermo Fisher, MA), anti-GFP antibody (GF28R) at 1:2000 dilution (Thermo Fisher, MA) and anti–AtpB antibody at 1:5000 dilution (Agrisera, Sweden) as a loading control. HRP-conjugated secondary antibody was used to detect signal with SuperSignal West Femto (Thermo Fisher, MA). Image was acquired with Image Quant LAS 4000 (GE Healthcare, IL). The band intensity was quantified with ImageJ (NIH).

### Indirect immunofluorescence

Indirect immunofluorescence was performed as described [35]. The primary antibodies are anti-mCherry monoclonal antibody (16D7) at 1:200 dilution (Thermo Fisher, MA) and anti-α-tubulin antibodies (clone B-5-1-2, Sigma-Aldrich) at 1:5000. The secondary antibody was anti-rat-Alexa 568 (Thermo Fisher, MA) at 1:1000 and DyLight 405 goat anti-mouse antibody (BioLegend, CA) at 1:2000, respectively. Cells were stained with 1 μM or 0.5 μM Sytox Green (Invitrogen, CA). Images were collected using an inverted Zeiss LSM 510 Laser Scanning confocal microscope. Images in S1 Fig were collected using DeltaVision microscope. Images were exported in TIFF format, merged and analyzed using ImageJ (NIH).

### Time-lapse microscopy

Cells arrested in G1 by nitrogen deprivation were transferred to agarose pads and imaged with a 63X objective on a Leica DMI6000B microscope with the objective and stage heated to 33°C. Pad architecture and other procedures were based on the budding yeast setup of Di Talia et al [44]. Images were acquired using custom software, as previously described for yeast timelapse [45], modified to adjust autofocus for *Chlamydomonas*. Deconvolution was employed to subtract the chloroplast-specific signal in the Venus channel. All images in a given movie were analyzed identically.

### Live cell imaging

Cells were observed under Nikon Eclipse 90i fluorescence microscope using a 60×/1.45 numerical aperture Plan Apochromatic objective lens (Nikon, Tokyo, Japan) fitted with an Intensilight Ultra High Pressure 130-W mercury lamp (Nikon, Tokyo, Japan). Images were captured with a Clara interline charge-coupled device camera (Andor, Belfast, United Kingdom). The images were aquired with NIS-Elements software (Nikon, Tokyo, Japan), and processed with ImageJ (NIH, Bethesda, MD).

### MCM4 loading assay

Cells were blocked in G1 by nitrogen starvation, then refed for 12 hours, then plasma membranes were permeabilized by 0.03% Triron-X and 0.03% Digitonin for 3 minutes. In S3C Fig, Ethidium Bromide at concentration of 10μg/ml was added to stain DNA after the membrane permeabilization. 0.25M of NaCl was further subsequently. The same method was used for Fig 9.

## Supporting information

S1 Fig Chlamydomonas plasmid construction. A universal mCherry plasmid map is shown. Tagging can be mCherry, Venus or GFP.
(PPTX)

S2 Fig Transformation of *CDC45* transgene into the corresponding temperature sensitive mutant. *CDC45-mCherry* plasmid was constructed and transformed into *cdc45* temperature sensitive mutant. Transformants were selected from plates at 21°C and aligned as 364 well format. The plates were incubated at 21 or 33°C for 10 days. The colony with red arrow was further analyzed. Empty plasmid was used as a negative control.
(PPTX)

S3 Fig DNA stayed in the nucleus after detergent and salt treatment. (A) *MCM4-Venus* in *rnr1-1*, *cdkb1-1*, *orc1-1*, *cdc6-1 or mcm6-2* cells were arrested in G1 and released at 33°C for 12 hrs. (B) Cells were stained with Ethidium Bromide. (C) Cells were further treated with 0.03% TritonX and 0.03% Digitonin to permeabilize membranes. (D) Cells were further treated with 0.25M NaCl.
(PPTX)

S4 Fig CDC6 and MCM2-7 with CDK and NLS sites. Blue box shows minimal CDK sites which contains S/T-P. Pink box shows conserved CDK sites with S/T-P-X-K/R. Red bar shows Phospho-Degron which contains S/T-P-X-X-S/T-P-X-K/R. Yellow box shows NLS with score using NLS Mapper (nls-mapper.iab.keio.ac.jp).
(PPTX)

S1 Table *C*. *reinhardtii* strains used in this paper. (XLSX)

S2 Table Oligonucleotides sequence used to verify the mutation. Lowercase letters indicate additional external sequence.
(DOCX)

S1 Movie *MCM4-Venus*. (MP4)

S2 Movie *MCM4-Venus cdc27-6*. (MP4)

S3 Movie *MCM4-Venus cdk1-1*. (MP4)

S4 Movie *MCM4-Venus cycb-5*. (MP4)

S5 Movie *Ble-GFP*. (MP4)

S6 Movie *Ble-GFP cdc27-6*. (MP4)
