# Supplementary figures and images for: Control of pre-replicative complex during the division cycle in Chlamydomonas reinhardtii
Source: PLoS Genet. 2021 Apr 28;17(4):e1009471. doi: 10.1371/journal.pgen.1009471 (PMC8081180; doi:10.1371/journal.pgen.1009471)

## Slide 1
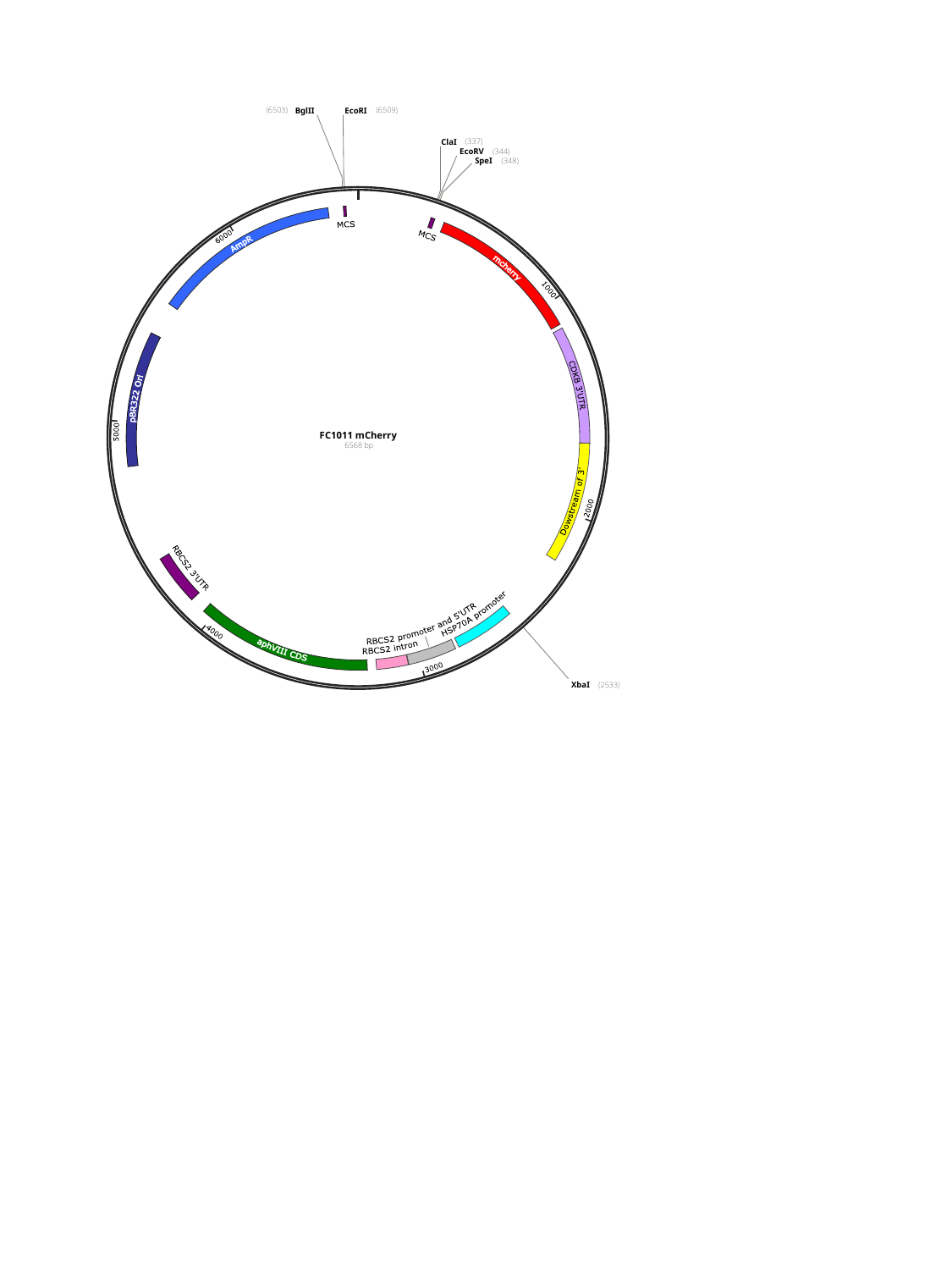

Supplement: S1 Fig — A universal mCherry plasmid map is shown. Tagging can be mCherry, Venus or GFP. (PPTX) [file pgen.1009471.s001.pptx]

## Slide 1
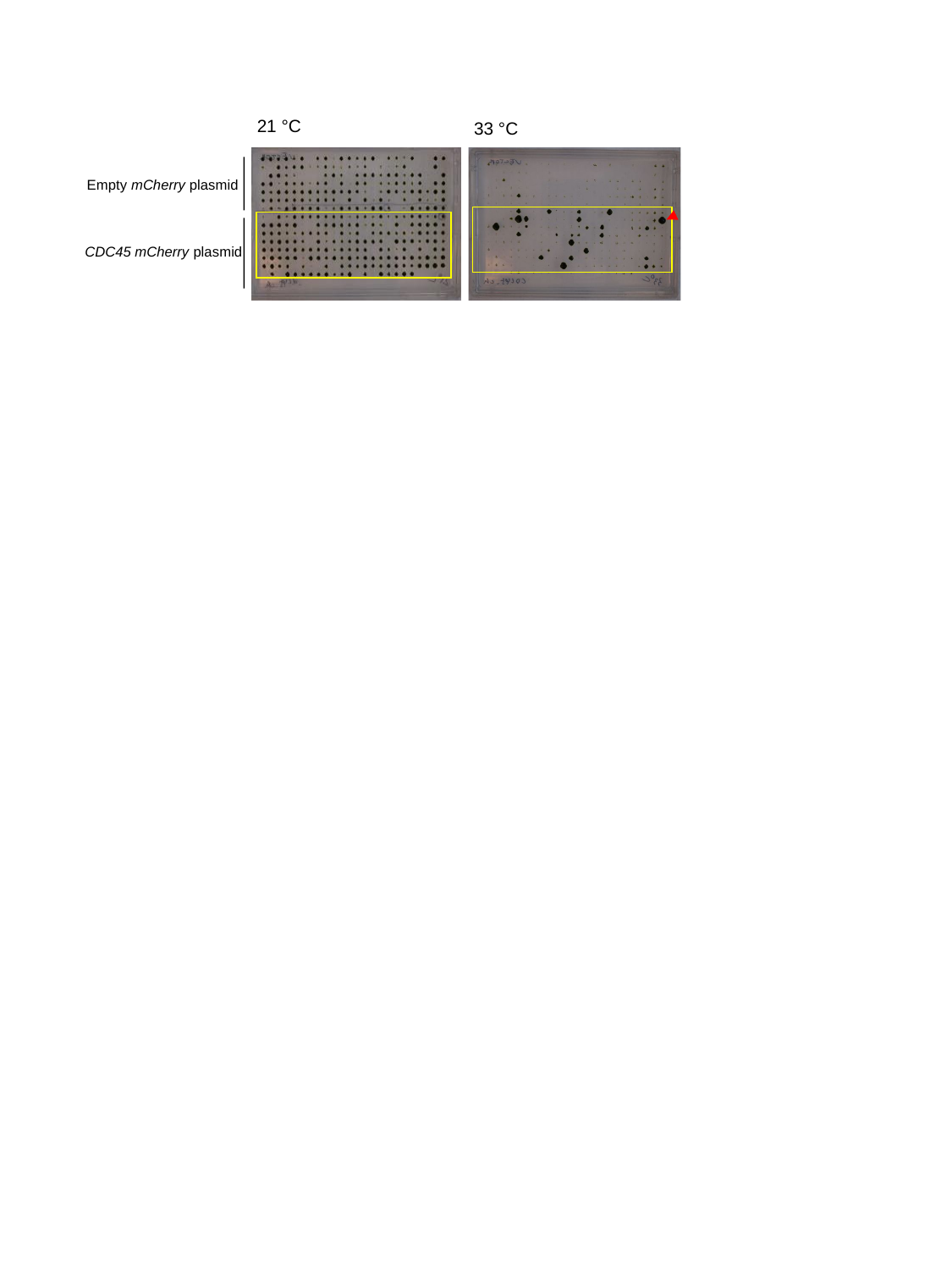

21 °C
33 °C
Empty mCherry plasmid
CDC45 mCherry plasmid

Supplement: S2 Fig — CDC45-mCherry plasmid was constructed and transformed into cdc45 temperature sensitive mutant. Transformants were selected from plates at 21°C and aligned as 364 well format. The plates were incubated at 21 or 33°C for 10 days. The colony with red arrow was further analyzed. Empty plasmid was used as a negative control. (PPTX) [file pgen.1009471.s002.pptx]

## Slide 1
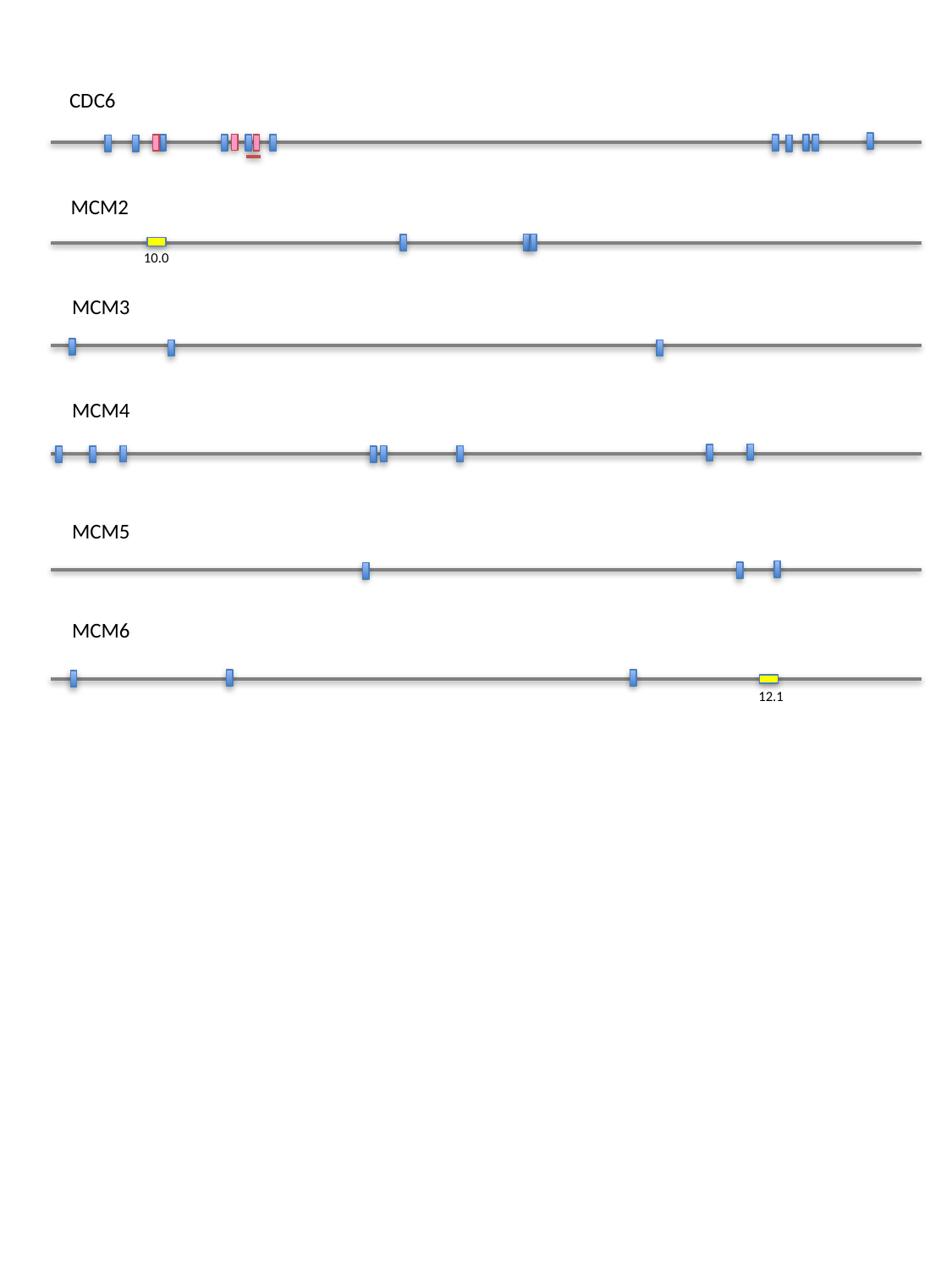

CDC6
MCM2
10.0
MCM3
MCM4
MCM5
MCM6
12.1

Supplement: S4 Fig — Blue box shows minimal CDK sites which contains S/T-P. Pink box shows conserved CDK sites with S/T-P-X-K/R. Red bar shows Phospho-Degron which contains S/T-P-X-X-S/T-P-X-K/R. Yellow box shows NLS with score using NLS Mapper (nls-mapper.iab.keio.ac.jp). (PPTX) [file pgen.1009471.s004.pptx]
