# Supplementary material for: Control of pre-replicative complex during the division cycle in Chlamydomonas reinhardtii
Source: PLoS Genet. 2021 Apr 28;17(4):e1009471. doi: 10.1371/journal.pgen.1009471 (PMC8081180; doi:10.1371/journal.pgen.1009471)
Supplement: S3 Fig — (A) MCM4-Venus in rnr1-1, cdkb1-1, orc1-1, cdc6-1 or mcm6-2 cells were arrested in G1 and released at 33°C for 12 hrs. (B) Cells were stained with Ethidium Bromide. (C) Cells were further treated with 0.03% TritonX and 0.03% Digitonin to permeabilize membranes. (D) Cells were further treated with 0.25M NaCl. (PPTX) [file pgen.1009471.s003.pptx]

## Slide 1
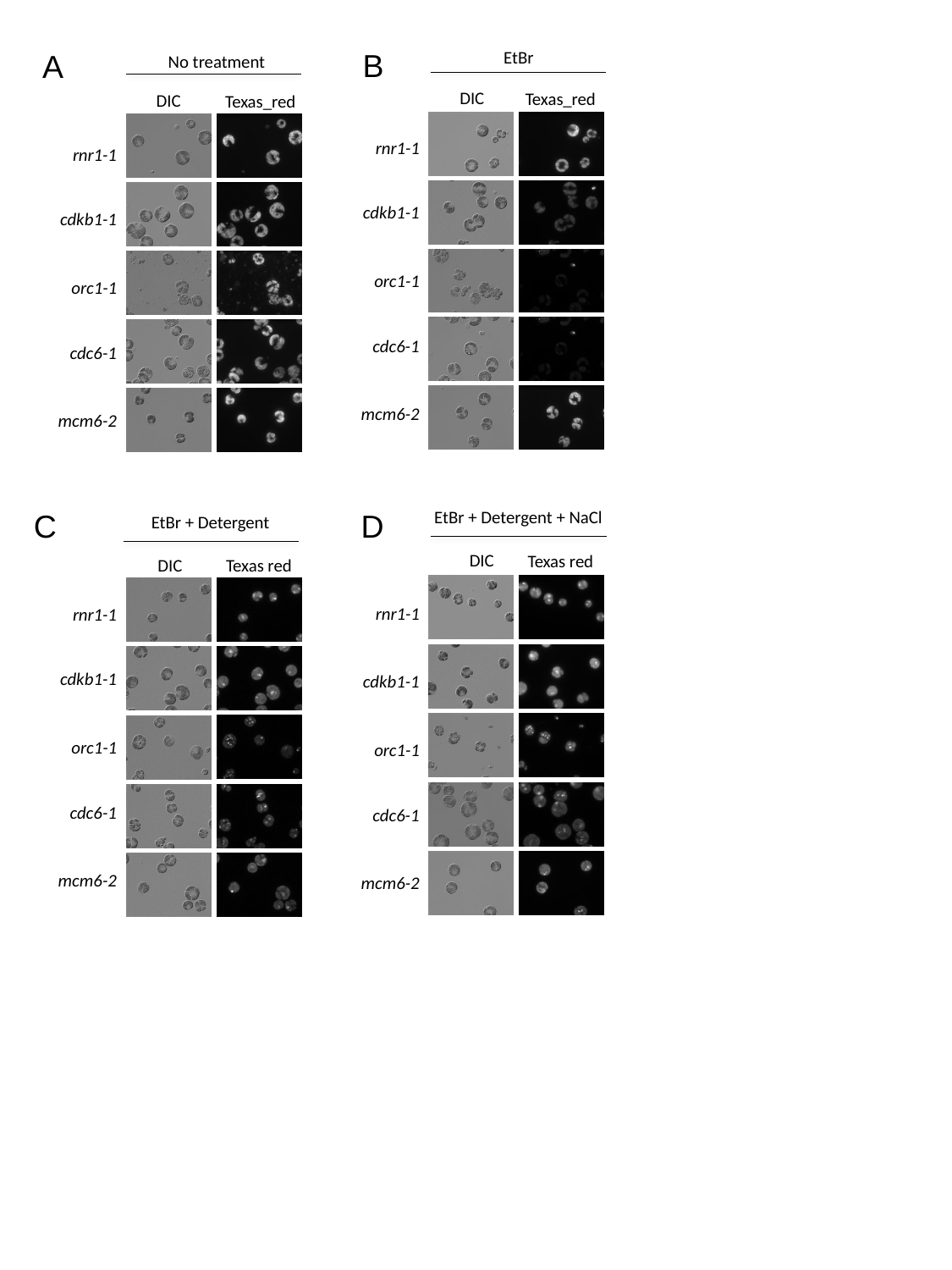

B
EtBr
A
No treatment
DIC
Texas_red
DIC
Texas_red
rnr1-1
cdkb1-1
orc1-1
cdc6-1
mcm6-2
rnr1-1
cdkb1-1
orc1-1
cdc6-1
mcm6-2
EtBr + Detergent + NaCl
D
C
EtBr + Detergent
DIC
Texas red
DIC
Texas red
rnr1-1
cdkb1-1
orc1-1
cdc6-1
mcm6-2
rnr1-1
cdkb1-1
orc1-1
cdc6-1
mcm6-2
