# Supplementary material for: Control of pre-replicative complex during the division cycle in Chlamydomonas reinhardtii
Source: PLoS Genet. 2021 Apr 28;17(4):e1009471. doi: 10.1371/journal.pgen.1009471 (PMC8081180; doi:10.1371/journal.pgen.1009471)
Supplement: S2 Table — Lowercase letters indicate additional external sequence. (DOCX) [file pgen.1009471.s006.docx]

Supplemental Table 2

| gene | primer sequence |
| --- | --- |
| orc1 | GCTCACCTGCAGCTGGTGATGCT |
|  | acttggataggggattatctGCTCACCTGCAGCTGGTGATACC |
|  | CCAGCGCTGTCCGCTCATGC |
|  |  |
| cdc6 | gccAGCGGCTGCGCGGCCGA |
|  | acttggataggggattatctgccAGCGGCTGCGCGGCGGT |
|  | TTCTCTGACATCACCAGGCA |
|  |  |
| mcm4 | CTCCGCCGCCTCGTCGCGCA |
|  | acttggataggggattatctCTCCGCCGCCTCGTCGCACC |
|  | GGCGCCGGCGACCTCATCTCG |
|  |  |
| mcm6-981A | GTAGGTCTCGTGCAGGTGCA |
|  | acttggataggggattatctGTAGGTCTCGTGCAGGTGCG |
|  | TTTCGGCGCCAGTACACGCA |
|  |  |
| mcm6-GHI | GGCGCAGAAGTTCAACTACGTC |
|  | acttggataggggattatctGCGCAGAAGTTCAACCACGAT |
|  | GATTGCAGGGATGCCTAGATGC |
|  |  |
| mcm6-GHI | GTCGGCGCAGAAGTTCAACTACATC |
|  | acttggataggggattatctGGCGCAGAAGTTCAACCACCAT |
|  | ACACGCTCCGCTCCCTCATG |
|  |  |
| mcm7 | GTCACCTCCTGGTACACCTC |
|  | acttggataggggattatctGTCACCTCCTGGTACACCTT |
|  | CGTGGCTGGTATTTGCAAAA |
|  |  |
| RFC1 | CATCAAGCGCAGCAAGAACC |
|  | acttggataggggattatctCATCAAGCGCAGCAAGAGCA |
|  | CGCTTGGAGATCTGCAGCAC |
|  |  |
| rnr1 | GGACGCCGCCAACCGCTTCT |
|  | acttggataggggattatctGGACGCCGCCAACAGCTTTC |
|  | GGCGCATGTTGGAGCGCTGC |
|  |  |
| zeu1 | GCTCCATGGGCGTGAAGACC |
|  | acttggataggggattatctGCTCCATGGGCGTGAAGGCT |
|  | GTGTCGGTGCGTGCAGGTG |
|  |  |
| cycb1 | GCTGTCCAGCTACCTCAACG |
|  | acttggataggggattatctGCTGTCCAGCTACCTCAGCA |
|  | GCAATCGGCCTTCTCGTACG |
|  |  |
| cdkb | ACCCTATGGTACCGCGCACC |
|  | acttggataggggattatctACCCTATGGTACCGCGCCCA |
|  | CCACCTTGCGCACGAGCTCAG |
|  |  |
| cdc20 | GGACGACTACTACCTCAACCTACT |
|  | acttggataggggattatctGGACGACTACTACCTCAACCTATC |
|  | TTCCCTTTCCTGCCCTTCAC |
|  |  |
| cdc27 | gttcccgcctcctctcccct |
|  | acttggataggggattatctTGCCACGTGGGTGGTGATCA |
|  | TGCCACGTGGGTGGTGATGG |
